# Dr. Wm. H. Barcroft

**Published:** 1912-05

**Authors:** 


Dr. Wm. H. Barcroft, of Coshockton, Ohio, a graduate of Starling (Columbus) Medical College, 1875, started to make a professional call on March 17, and was found frozen to death in field, March 23. He was 59 years old. Here are two phases of medical heroism and martrydom, as different as possible in detail, identical in spirit, equal in honor and value, if not in glory.

